# Theaflavins Improve Memory Impairment and Depression-Like Behavior by Regulating Microglial Activation

**DOI:** 10.3390/molecules24030467

**Published:** 2019-01-28

**Authors:** Yasuhisa Ano, Rena Ohya, Masahiro Kita, Yoshimasa Taniguchi, Keiji Kondo

**Affiliations:** Research Laboratories for Health Science & Food Technologies, Kirin Company Ltd., Kanazawa-ku, Yokohama-shi, Kanagawa 236-0004, Japan; Rena_Ohya@kirin.co.jp (R.O.); Masahiro_Kita@kirin.co.jp (M.K.); Yoshimasa_Taniguchi@kirin.co.jp (Y.T.); kondok@kirin.co.jp (K.K.)

**Keywords:** black tea polyphenol, depression, inflammation, memory, microglia, neuroprotection, theaflavins

## Abstract

Inflammation in the brain is associated with various disorders including Alzheimer’s disease and depression. Thus, inflammation has received increasing attention regarding preventive approaches to such disorders. Epidemiological investigations have reported that drinking tea reduces the risk of dementia and depression. Theaflavins, a polyphenol found in black tea, are known to have anti-oxidative and anti-inflammation effects, but the effects of theaflavins on cognitive decline and depression induced by inflammation have not been investigated. To address this research gap, the present study assessed whether theaflavins could protect synapses and dendrites damaged by inflammation and prevent concomitant memory impairment and depression-like behavior in mice. Intracerebroventricular injection with lipopolysaccharide (LPS) induces neural inflammation associated with reduced spontaneous alternations in the Y-maze test and increased immobility in the tail suspension test, indicating impaired spatial memory and depression-like behavior, respectively. Oral administration with theaflavins prevented these behavioral changes induced by LPS. Theaflavins also suppressed productions of inflammatory cytokines and prevented dendritic atrophy and spine loss in the brain. Notably, theaflavins have a stronger anti-inflammatory effect than other polyphenols such as catechin, chlorogenic acid, and caffeic acid. These results suggest that theaflavins can suppress neural inflammation and prevent the symptoms of inflammation-related brain disorders.

## 1. Introduction

Inflammation in the brain is associated with various brain disorders and may accelerate their pathologies [1,2,3,4]. Recent studies have revealed the involvement of microglia, which regulate immune response and inflammation in the brain [5], in brain disorders. Microglia play a key role in maintaining the neuroenvironment by removing pathogens, waste products, and old synapses via phagocytosis and by promoting synapse extension [6]. These functions of microglia decline due to various factors including aging and stress [7,8]. In the brains of patients with Alzheimer’s disease and depression, microglia are excessively activated and produce reactive oxygen species and inflammatory cytokines that can damage neurons, leading to cognitive decline [9] and psychiatric syndromes [10]. Regulation of neuroinflammation and microglia are, thus, of increasing interest for prevention and as therapeutic targets [11,12].

Epidemiological studies have reported that the consumption of tea has preventive effects on psychiatric disorders, including depression [13] and cognitive decline leading to dementia [14,15]. An epidemiological survey in Finland reported that people who consume black tea score lower on the Beck Depression Inventory and have lower rates of depression [16], whereas another epidemiological study reported that tea reduces the risk of anxiety and depressive mood [17]. Higher tea consumption is also associated with a significant reduction in the risk of cognitive disorders [14,15]. Moreover, a French cohort showed that the intake of a combination of polyphenols from specific plant products—including tea and red wine—was associated with lower dementia risk [14].

In the case of green tea, polyphenols such as epicatechin, epigallocatechin, and epigallocatechin-3-gallate (EGCG) may be beneficial compounds. Recent reports have shown that green tea polyphenol improves cognitive decline and depression in preclinical studies [18,19]. In an experiment using obese mice model, EGCG improved high-fat- and high-fructose-induced cognitive impairment via regulation of the ERK/CREB/BDNF pathway [20]. An experiment using lipopolysaccharide (LPS)-induced memory-impaired mice showed that EGCG prevented the production of pro-inflammatory cytokines and memory impairment [21]. Black tea is the most widely consumed tea worldwide and is rich in polyphenol compounds, especially theaflavins. Theaflavins are antioxidant polyphenols with a reddish color formed by the condensation of flavan-3-ols in tea leaves during the fermentation of black tea [22,23]. Theaflavins are composed of theaflavin (TF1), theaflavin-3-monogallate (TF2a), theaflavin-3′-monogallate (TF2b), and theaflavin-3,3′-digallate (TF3). Theaflavins are reported to have anti-hyperglycemia [24], anti-inflammation [25], anti-viral [26,27], and anti-inflammatory activities [25]. On the other hand, the effects of theaflavins administered orally on cognitive decline and depression-like behavior induced by inflammation have not been investigated. To address this research gap, the present study assessed whether theaflavins administered orally could protect synapses and dendrites damaged induced by inflammation and prevent concomitant memory impairment and depression-like behavior in mice. In addition, the effects of theaflavins on primary microglia and neuronal cells were evaluated.

## 2. Results

### 2.1. Effects of Theaflavins on Memory Impairment and Depression Induced by Inflammation

**Figure 1:**
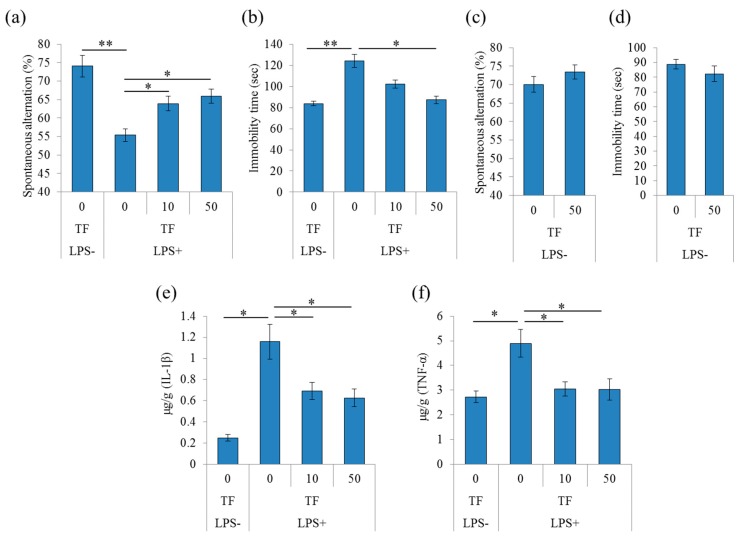
Effects of theaflavins on cognitive decline and depression-like behavior induced by lipopolysaccharide (LPS). (**a**–**d**), Crl:CD1 mice were orally administered 0, 10, or 50 mg/kg of theaflavins (TF) for 3 days and intracerebroventricularly injected with PBS or 10 μg LPS 1 h after the last administration. The mice were subjected to the spontaneous alternation test and tail suspension test at 1 day and after LPS injection. Spontaneous alternations and arm entries in the Y-maze to evaluate spatial memory (**a**); immobility time in the tail suspension test 1 day after LPS (**b**). Crl:CD1 mice were orally administered 0 or 50 mg/kg of theaflavins (TF) for 3 days and subjected to the spontaneous alternation test (**c**) and tail suspension test (**d**). (**e**,**f**), Crl:CD1 mice were orally administered 0, 10, or 50 mg/kg of TF for 3 days and intracerebroventricularly injected with phosphate buffered saline (PBS) or 10 μg LPS 1 h after the last administration. Amounts of IL-1β (**e**) and tumor necrosis factor (TNF)-α (**f**) in the hippocampus 24 h after LPS injection, respectively. Data are mean ± SE of 10 mice per group. The *p* values shown were calculated using the Student’s *t*-test (LPS [−] vs. [+] at 0 mg/kg TF) and one-way ANOVA followed by Dunnett’s test (LPS [+] at 0 mg/kg TF vs. LPS [+] at 10 and 30 mg/kg TF). * *p* < 0.05 and ** *p* < 0.01.

To evaluate the effects of theaflavins on memory impairment induced by inflammation, mice received an injection with LPS to the lateral ventricle of the brain and were subjected to the Y-maze test. LPS treatment significantly reduced spontaneous alternations in the Y-maze, indicating impairment in spatial working memory compared with phosphate buffered saline (PBS) treatment indicated as LPS(-) (Figure 1a). LPS did not change arm entries (data not shown). Administration theaflavins once daily for 3 days significantly attenuated the LPS-induced reduction in spontaneous alternations compared with the control treatment (Figure 1a). Next, to evaluate the effects of theaflavins on depression-like behavior, mice with LPS injection to the lateral ventricle of the brain were subjected to the tail suspension test (TST). LPS significantly increased the immobility time during suspension, indicating that LPS induced a depression-like behavior (Figure 1b). Theaflavins administration significantly reduced the immobility time compared with the control treatment (Figure 1b). These results indicate that theaflavins improved memory impairment and depression-like behavior induced by LPS. On the other hand, theaflavins did not change the spontaneous alternation of Y-maze test and immobility time of TST in mice without the injection with LPS (Figure 1c,d, respectively).

To evaluate the effects of theaflavins on inflammation induced by the LPS injection to the brain, the amounts of cytokines and chemokines in the hippocampus were measured by a multiplex system. The amounts of tumor necrosis factor (TNF)-α and IL-1β in the hippocampus of LPS-treated mice were significantly increased compared with those in the PBS-treated mice (Figure 1e,f, respectively). Furthermore, administration of theaflavins reduced the amounts of TNF-α and IL-1β in the LPS-treated mice (Figure 1e,f, respectively).

**Figure 2:**
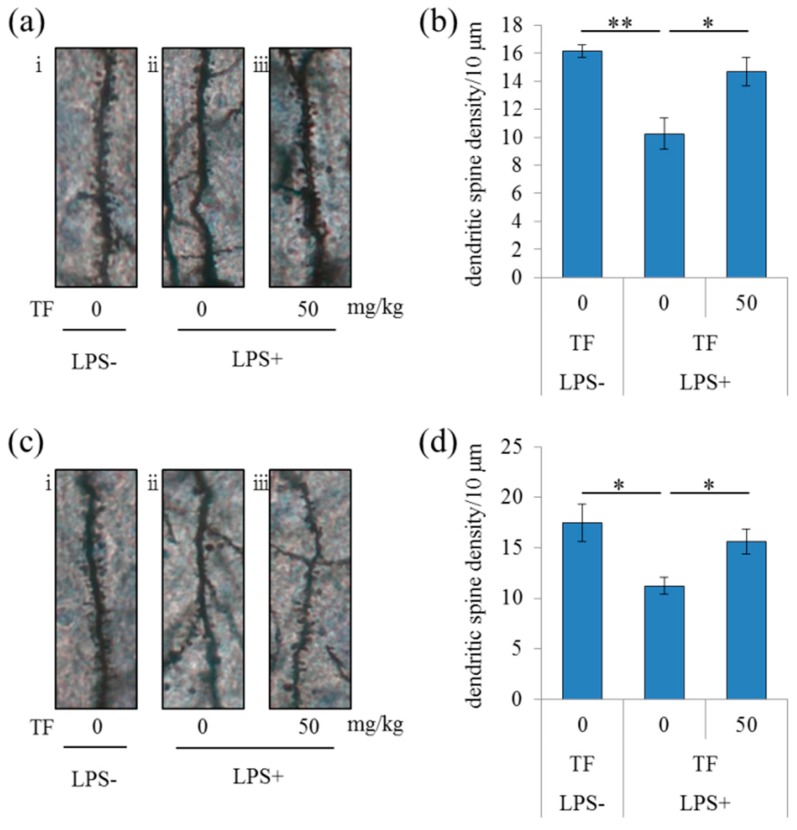
Effects of theaflavins on LPS-induced changes in dendritic spine density. Crl:CD1 mice were orally administered 0 or 50 mg/kg of theaflavins (TF) for 3 days and intracerebroventricularly injected with PBS or 0.3 mg/kg LPS 1 h after the last administration. The brain was subjected to Golgi staining 1 day after LPS injection. (**a**,**c**) Representative photomicrographs of Golgi-stained neurons in the CA1 of the hippocampus (a-i, ii, iii) and prefrontal cortex (c-i, ii, iii) of mice without LPS, with LPS and 0 mg/kg TF, with LPS and 50 mg/kg TF, respectively. Number of dendritic spines per 10 μm in the CA1 (**b**) and prefrontal cortex (**d**). Data are mean ± SE of 10 mice per group. The *p* values shown were calculated using the Student’s *t*-test. * *p* < 0.05 and ** *p* < 0.01.

To evaluate the effects of theaflavins on neural dendrites in LPS-treated mice, dendrites were analyzed by Golgi staining. LPS injection to the brain significantly reduced the number of apical dendrites of pyramidal neurons and spines along those dendrites in the CA1 region of the hippocampus compared with the control treatment, but this effect of LPS was significantly prevented by treatment with theaflavins (Figure 2a,b). This theaflavins-induced prevention from the LPS-induced dendritic atrophy and spine loss was also observed in the prefrontal cortex (Figure 2c,d). These results suggest that administration of theaflavins reduced LPS-induced inflammation in the brain and the concomitant dendritic atrophy of pyramidal neurons in the hippocampus and prefrontal cortex.

### 2.2. Effects of Theaflavins on the Inflammatory Response of Cultured Microglia

**Figure 3:**
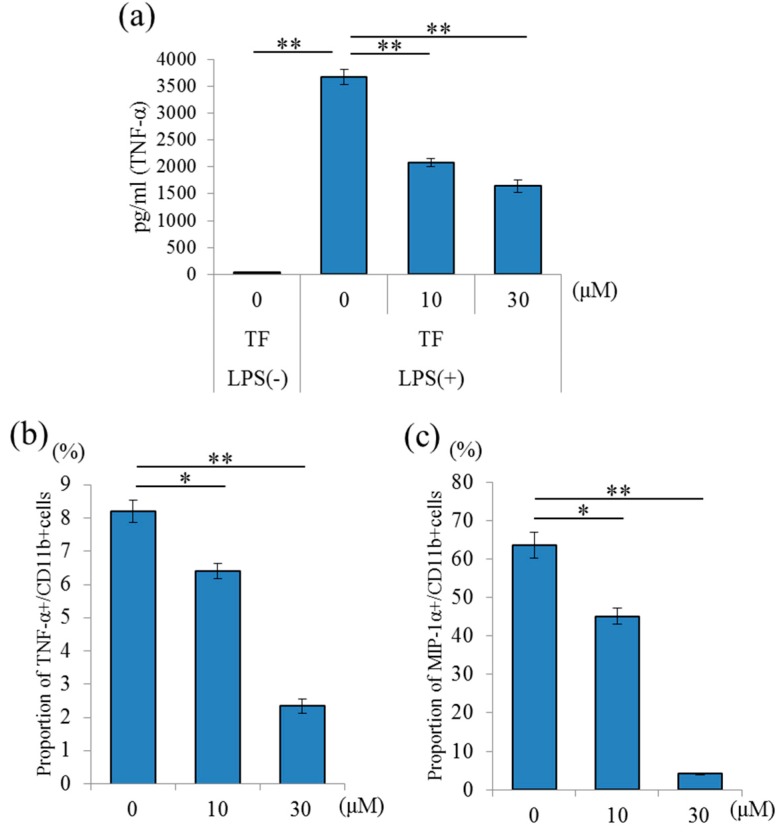
Effects of in vitro theaflavins (TF) treatment on microglial anti-inflammatory activity. (**a**), Amount of TNF-α in the supernatant of microglia pretreated with 0, 10, or 30 μM TF and treated with 5 ng/mL LPS and 0.5 ng/mL IFN-γ. (**b**,**c**) Intracellular cytokine production in microglia pretreated with 0, 10, or 30 μM TF and treated with a leukocyte activation-cocktail with BD GolgiPlug. Scatter plots and percentages of macrophage inflammatory proteins (MIP)-1α- and TNF-α-producing cells in CD11b-positive cells, respectively. Columns and bars represent the means and SEs of triplicate wells per sample, respectively. The *p* values shown were calculated using the Student’s *t*-test (LPS [−] vs. [+] at 0 μM TF) and one-way ANOVA followed by Dunnett’s test (LPS [+] at 0 μM TF vs. LPS [+] at 10 and 30 μM TF). * *p* < 0.05 and ** *p* < 0.01.

To evaluate the effects of theaflavins on primary microglia, the levels of cytokines and chemokines in the supernatant of microglial culture treated with LPS were quantified by enzyme-linked immune sorbent assay (ELISA) or a multiplex system. Theaflavins at a concentration of 10 to 30 μM significantly reduced TNF-α production in the supernatant of a microglial culture treated with LPS (Figure 3a) without apparent cytotoxicity or cell proliferation (data not shown). Next, to measure cytokine production, the percentages of TNF-α- and macrophage inflammatory proteins (MIP)-1α-producing cells in CD11b-positive microglia were analyzed using flow cytometry. Treatment with theaflavins at 10 and 30 μM significantly reduced the percentages of TNF-α- and MIP-1α-producing microglia compared with the control treatment (Figure 3b,c, respectively). This result suggests that theaflavins suppressed inflammatory responses of microglia.

### 2.3. Theaflavins-Induced Suppression of Neurotoxic Effects of Activated Microglia

**Figure 4:**
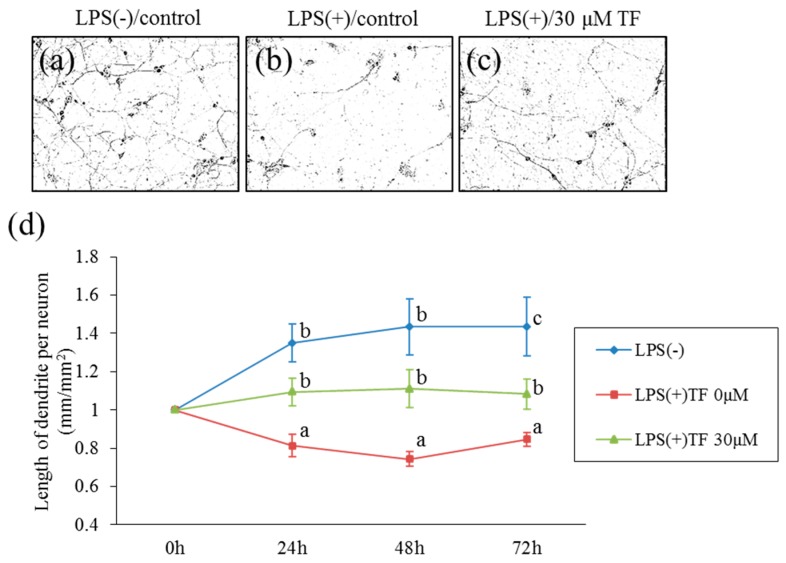
Effects of the conditioned medium of microglial culture treated with theaflavins on neuroprotection. (**a**–**c**), Morphological changes of the neuronal dendrites in Neuro-2A cells. Differentiated Neuro-2A were cultured with the supernatant of microglia with PBS as a vehicle (**a**) and with LPS and interferon (IFN)-γ after pretreatment with 0 or 30 μM theaflavins (**b**,**c**, respectively). (**d**), Length of the dendrites per neuron (mm/mm^2^) after 0–72 h of culture. Bars represent the SE of three wells per sample. The *p* values shown were calculated using the Student’s *t*-test (LPS [−] vs. [+] at 0 μM TF) and one-way ANOVA followed by Dunnett’s test (LPS [+] at 0 μM TF vs. LPS [+] at 30 μM TF). Different letters indicate significant differences between groups (*p* < 0.05).

To examine whether theaflavins attenuate the neurotoxic effects of substances released from LPS-stimulated microglia differentiated neuronal, Neuro2A cells were cultured with a conditioned medium of microglia cultured with LPS. The lengths of neurites are shown in Figure 4a–c. A conditioned medium of LPS-stimulated microglia significantly decreased the length of neurites, whereas this effect was not observed with a conditioned medium of non-stimulated microglia (Figure 4d). Treatment of microglia with theaflavins before LPS stimulation attenuated the reduction in neurite length induced by a conditioned medium of LPS-stimulated microglia (Figure 4d). Direct treatment of Neuro2A cells with theaflavins at 30 μM had no effects on the growth of neurites (data not shown). These results show that theaflavins attenuated the neurotoxic effect of LPS-stimulated microglia.

**Figure 5:**
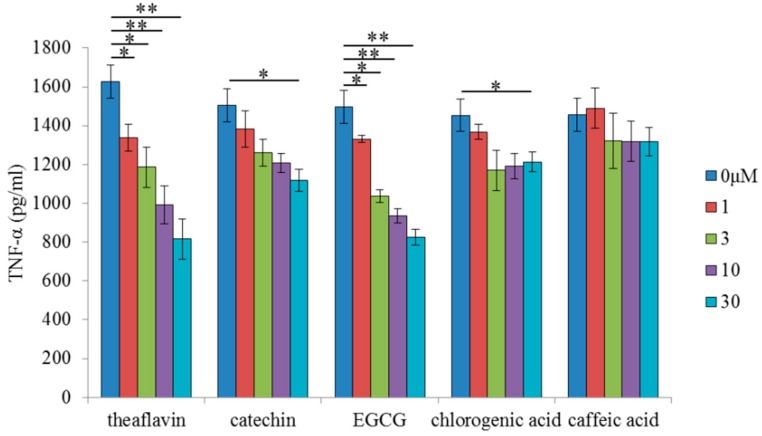
Effects of in vitro treatment with polyphenols on microglial anti-inflammatory activity. Amount of TNF-α in the supernatant of microglia pretreated with 0, 1, 3, 10, or 30 μM theaflavins, catechin, epigallocatechin-3-gallate (EGCG), chlorogenic acid, or caffeic acid and treated with 5 ng/mL LPS and 0.5 ng/mL IFN-γ. Columns and bars represent the means and SEs of three wells per sample, respectively. *p*-values shown in the graph were calculated by one-way ANOVA followed by the Dunnett’s test. * *p* < 0.05 and ** *p* < 0.01.

To compare the anti-inflammatory activity of theaflavins with those of other polyphenols, microglia were treated with theaflavins, catechin, EGCG, chlorogenic acid, or caffeic acid at concentrations of 0, 1, 3, 10, and 30 μM before LPS treatment. Pretreatment with theaflavins, catechin, and EGCG decreased LPS-induced production of TNF-α of microglia in a concentration dependent manner, whereas pretreatment with chlorogenic acid and caffeic acid had marginal effects (Figure 5). Theaflavins and EGCG reduced LPS-induced production of TNF-α to similar levels, whereas the effect was smaller with catechin. These results indicate that the anti-inflammatory effects of theaflavins on microglia are stronger than those of common polyphenols, but comparable to those of EGCG.

## 3. Discussion

The present study showed that the black tea polyphenol theaflavins suppress inflammatory responses in the brain and attenuate concomitant cognitive impairment and depression-like behavior. Theaflavins suppressed production of pro-inflammatory cytokines in murine and human microglia, and hence neurotoxic effects of activated microglia. This finding is consistent with epidemiological studies reporting that tea consumption suppresses cognitive decline and depressive symptoms [13,14,15]. The anti-inflammatory effect of theaflavins was stronger than those of other polyphenols like catechin in green tea and chlorogenic acid and caffeic acid in coffee, but it was equivalent to that of EGCG in green tea. Since EGCG has been reported to suppress pro-inflammatory cytokines in the brain and depression-like behavior in mice [21], the effects of theaflavins and EGCG may be comparable.

Inflammation in the brain is associated with cognitive dysfunction and psychiatric conditions as well as motor disorders and chronic fatigue [28]. In the present study, LPS injection to the brain induced dendritic atrophy of pyramidal neurons in prefrontal cortex and hippocampus, and pretreatment with theaflavins reduced the dendritic atrophy. Dendritic atrophy is associated with cognitive decline [29] and depression [30]. It has been reported that intracerebroventricular administration of LPS induces dendritic changes in the hippocampus and prefrontal cortex and depressive-like behavior, whereas suppression of inflammation can improve these impairments [31,32]. Because the hippocampus and prefrontal cortex are involved in spatial working memory [33] and depression [34], the neuroprotective effects of theaflavins in these areas may attenuate cognitive impairment and depression. Consistently, pretreatment of microglia with theaflavins attenuated neurotoxic effects of a conditioned medium of LPS-stimulated microglia, as measured by neurite shortening. It has also been reported that microglia activated by LPS produce large amounts of reactive oxygen species (ROS) and pro-inflammatory cytokines, leading to neuronal damage [35]. It is reported that theaflavins inhibit IκB kinase (IKK) and nuclear factor-κB (NF-κB) activation, which is supposed to suppress the inflammatory response of microglia against LPS stimulation [36]. Taken together, these findings support the neuroprotective effects of theaflavins through attenuating inflammation in the brain. 

Theaflavin is supposed not to enter into the brain via blood–brain barrier, because in case of EGCG, almost EGCG was detected in the intestine and blood, and EGCG was detectable in the brain, but the level in the brain was much lower than other organs [37]. This report suggests that theaflavins display the anti-inflammatory effects in the intestine and blood, but not directly in the brain. On the other hand, in the present study, we used the model mice injected with LPS. Acute brain inflammation damages the blood–brain barrier and more peripheral monocytes and microglia infiltrate into the brain [38,39]. Especially, it is reported that infiltrated neutrophils and monocytes rather than resident microglia are major inflammatory cells in LPS-injected brain [40]. In the LPS-injected model mice, theaflavin itself or monocyte affected by theaflavin in the peripheral tissue might be delivered to the brain. Further study needs to evaluate the blood–brain permeability in the normal mice and mice with brain inflammation. 

This study is subject to several limitations. The amount evaluated in the present study (10 mg/kg) is equivalent to 486 mL of tea for a human weighing 60 kg according to the following calculation. This amount is a little more than the typical daily intake. Based on the formula human equivalent dose (HED) (mg/kg) = Animal does (mg/kg) × (Animal Km/Human Km), where Human Km = 37, Mouse Km = 3 [41,42], mouse dose 10 mg/kg equals to human 0.81 mg/kg, that is, 48.6 mg theaflavins for a human weighing 60 kg. As tea contains approximately 100 mg/L theaflavins [43,44], 48.6 mg theaflavins is equivalent to 486 mL of tea for a human weighing 60 kg. However, as LPS treatment in the brain induces acute inflammation that is more severe than that in chronic inflammatory diseases such as dementia, the effective amount of theaflavins for memory impairment and depression-like behavior may be less than that used in the present study. Further research is needed to evaluate the effective amount of theaflavins for chronic inflammatory diseases. In addition, we need to analyze in the further study whether theaflavins enter the brain through the blood–brain barrier or act on the vagal nerve, leading to prevention from cognitive decline and depression.

Overall, our findings of the neuroprotective effects of theaflavins are consistent with previous epidemiological studies suggesting that consuming tea is beneficial for preventing cognitive decline and depression. Further study will elucidate the effects of theaflavins on animal models or patients of chronic diseases.

## 4. Materials and Methods

### 4.1. Preparation of Pure Theaflavins

**Figure 6:**
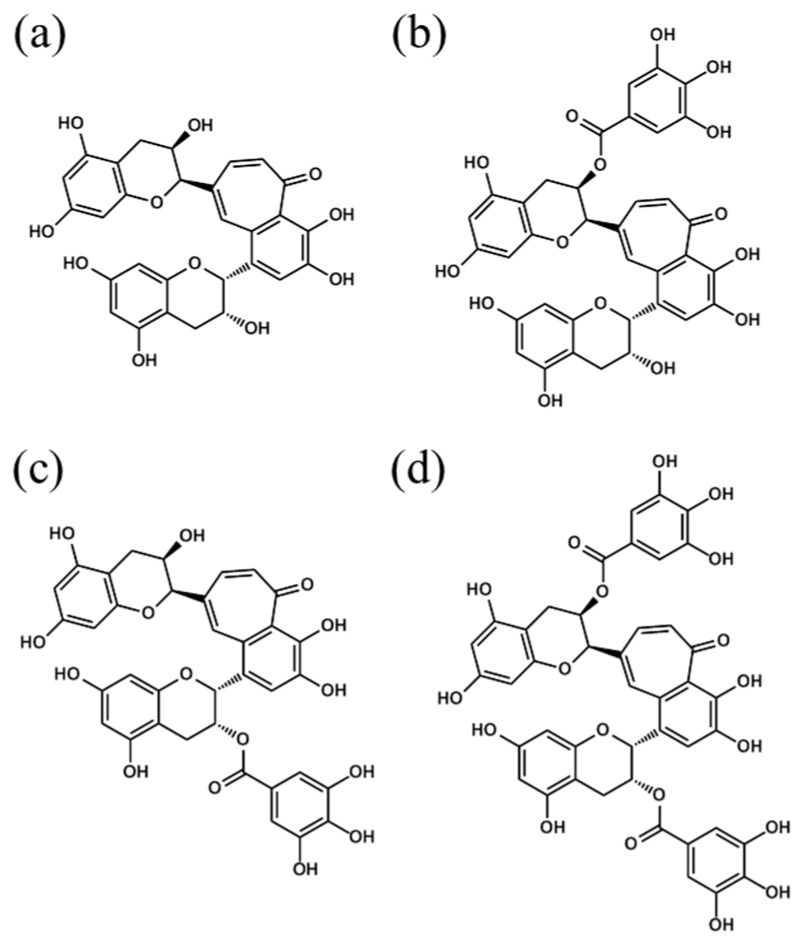
Chemical structures of theaflavins. Structures of theaflavin (TF1) (**a**), theaflavin 3-O-gallate (TF2a) (**b**), theaflavin 3′-O-gallate (TF2b) (**c**) and theaflavin 3,3′-O-digallate (TF3) (**d**). This is a figure, Schemes follow the same formatting. If there are multiple panels, they should be listed as: (**a**) Description of what is contained in the first panel; (**b**) Description of what is contained in the second panel.

TF40, a crude theaflavins extract containing about 40% *w*/*w* theaflavins, was purchased from Yaizu Suisankagaku Industry (Shizuoka, Japan). After being dissolved in 50% EtOH, TF40 (20 g) was repeatedly subjected to reversed-phase preparative high-performance liquid chromatography (HPLC; column: 150 × 22 mm id, 5 μm, Alltima C18 column; Systech, Tokyo, Japan); solvent: H_2_O/H_3_PO_4_ (85%), 100/1 (*v*/*v*) (solvent A) and acetonitrile (solvent B), linear gradient from 20% to 70% B; flow rate: 22.8 mL/min. Each fraction containing TF1, TF2a, TF2b, and TF3 described in Figure 6 was pooled and combined before acetonitrile was evaporated using a rotary evaporator. Next, theaflavins were extracted with ethyl acetate from the residual aqueous solution. and the organic solvent was evaporated. The residue was dissolved in a small amount of EtOH and added to water. The resulting suspension was lyophilized, yielding an orange, uniform powder (5.7 g). The purity of the theaflavins estimated by HPLC was more than 97%. Purified TFs were composed of 1.0% of TF1, 36.9% of TF2a, 14.7% of TF2b, and 44.6% of TF3.

### 4.2. Animals

Pregnant C57BL/6J mice and six-week-old Crl:CD1(ICR) male mice were purchased from Charles River Japan (Tokyo, Japan) and maintained at the Kirin Co. Ltd. All experiments were approved by the Animal Experiment Committee of Kirin Co. Ltd. and conducted in strict accordance with their guidelines in 2016–2017. The Approved ID was AN10339-Z00 and AN10623-Z00. All efforts were made to minimize the suffering. The mice were fed a standard rodent diet (CE-2; CLEA Japan, Tokyo, Japan) and maintained at room temperature (23 ± 1 °C) under a constant 12-h light/dark cycle (light period from 8:00 a.m. to 8:00 p.m.). 

### 4.3. Neural Inflammation Induced by LPS

Crl:CD1 mice were orally administered 0, 10, or 50 mg/kg of theaflavins dissolved in distilled water (10 mL/kg) once a day for 3 days. One hour after the last administration, the mice were deeply anesthetized with sodium pentobarbital (Kyoritsu Seiyaku, Tokyo, Japan) and intracerebroventricularly injected with 10 μg of LPS (L7895; Sigma-Aldrich, St. Louis, MO, USA), in accordance with previous work [45]. Briefly, LPS dissolved in PBS or PBS (for sham-operated controls) was injected into the cerebral ventricle as previously described [45]. Briefly, a micro-syringe with a 27-gauge stainless steel needle, 2 mm in length, was used for the microinjection. The needle was inserted unilaterally 1 mm to the right and left of the midline point equidistant from each eye in both left and right hemispheres, at an equal distance between the eyes and ears, and perpendicular to the plane of the skull (anteroposterior, −0.22 mm from the bregma; lateral). LPS was delivered gradually within 30 s. The needle was withdrawn after waiting 30 s. Twenty-four hours later, the hippocampus and frontal cortex of the corresponding hemisphere were homogenized in Tris-buffered saline buffer (Wako, Tokyo, Japan) containing a protease inhibitor cocktail (BioVision, Milpitas, CA, USA) with a multi-beads shocker (Yasui Kikai, Osaka, Japan). After centrifugation at 50,000× *g* for 20 min, the supernatants were collected. The total protein concentration of each supernatant was measured using a bicinchoninic acid (BCA) protein assay kit (ThermoScientific, Yokohama, Japan). To evaluate inflammation in the brain, the amounts of cytokines and chemokines in the supernatants were quantified using a multiplex system (Biopoex; Bio-Rad, Hercules, CA, USA). The other hemisphere was used for morphological analysis of the dendrites. Brain sections at the bregma −2.06 mm were prepared and stained using the FD Rapid GolgiStain Kit (FD Neuro Technologies, Columbia, MD, USA) following the manufacturer’s instructions. Spines were counted within the CA1, and the prefrontal cortex dendrites were counted starting from their point of origin from the primary dendrite, as previously described [32]. For spine density measurements, all areas containing 50 to 100 μm of secondary dendrites from the neuron were used.

In the experiment evaluating cognitive function and depression, mice were orally administered 0, 10, or 50 mg/kg theaflavins daily for 3 days, and 1 h after the last administration, mice were intracerebroventricularly injected with 10 μg of LPS. Twenty-four hours after LPS treatment, the mice were subjected to the tail suspension test (TST) and the spontaneous alternation test, as described below.

### 4.4. Tail Suspension Test

The TST was used to assess behavioral despair, which is a feature of depression. Each mouse was individually suspended by its tail using adhesive tape in a box for 6 min while being video recorded. The video was carefully scored for total time of immobility during suspension. Mice were considered immobile only when they hung passively and were completely motionless.

### 4.5. Spontaneous Alternation Test

The spontaneous alternation test was performed as previously described [46,47]. A three-arm Y-maze (25 cm long × 5 cm wide × 20 cm high) with equal angles between all arms constructed from dark black polyvinyl plastic was used. Each mouse was initially placed in one arm, and the sequence and number of arm entries were counted for 8 min. The alternation score (%) for each mouse was defined as the ratio of the actual number of alternations to the possible number (defined as the total number of arm entries minus two) multiplied by 100, i.e., % Alternation = [(Number of alternations)/(Total arm entries − 2)] × 100. 

### 4.6. Primary Murine Microglia Cell Culture

Primary microglial cells were isolated from the brains of newborn C57BL/6J mice (<7 days old) via magnetic cell sorting after conjugation with anti-CD11b antibodies (Miltenyi Biotec, Bergisch Gladbach, Germany), as previously described [48]. The isolated CD11b-positive cells (>90% pure as evaluated by flow cytometry) were plated in poly-D-lysine (PDL)-coated 96-well plates (BD Biosciences, Billerica, MA, USA) and cultured in DMEM/F-12 (Gibco, Carlsbad, CA, USA) medium supplemented with 10% fetal bovine serum (FBS; Gibco) and 100 U/mL penicillium/streptomycin (Sigma-Aldrich). Microglia isolated from newborn C57BL/6J mice were plated at a density of 30,000 per well in a PDL-coated plate. 

To measure inflammatory cytokine production in the supernatant, microglia were treated with theaflavins, catechin (Sigma-Aldrich), EGCG (Sigma-Aldrich), chlorogenic acids (Sigma-Aldrich), or caffeic acid (Sigma-Aldrich) for 12 h, and then treated with LPS (5 ng/mL; Sigma-Aldrich) and interferon-γ (IFN-γ, 0.5 ng/mL; R&D systems, Minneapolis, MN, USA) for 12 h. After stimulation, the supernatants were assayed using a Bio-Plex assay system (Bio-Rad).

To measure intracellular cytokines, microglia were treated with the test samples for 12 h, then treated with a leukocyte activation-cocktail with BD GolgiPlug^TM^ (BD Biosciences, San Jose, CA, USA) for 12 h, and then assessed using the BD Cytofix/Cytoperm Fixation/Permeabilization kit (BD Biosciences) as described in the previous study [49]. The cells were then stained with the anti-MIP-1α-PE (eBioscience, San Diego, CA, USA), anti-TNF-α-FITC (eBioscience), and anti-CD11b-APC-Cy7 (BD Biosciences) antibodies. The cells were finally analyzed with a flow cytometer (FACSCantoII; BD Biosciences). Each sample was assayed in three wells.

### 4.7. Neurotoxicity Assay

To evaluate the effects of the microglial culture supernatant treated with theaflavins and LPS, a neurotoxicity assay was conducted as described in our previous work [50]. The mouse neuroblastoma Neuro2A cell line (ATCC CCL-131) was maintained in minimum essential medium (MEM, Gibco) medium supplemented with 10% FBS (Gibco), non-essential amino acids (Gibco), and 100 U/mL penicillin/streptomycin (Sigma-Aldrich). Neuro2A cells were plated at a density of 4000 cells per well in a 96-well cell culture plate (Essen Bioscience, Welwyn Garden City, UK). After 24 h, FBS was added at a final concentration of 1% along with all-trans retinoic acid (Wako) to a final concentration of 10 μM to induce the Neuro2A to differentiate into neuronal cells. After removing the medium, the cells were incubated with microglial culture supernatant. Microglial culture supernatants were prepared as follows: first, microglia from newborn C57BL/6J mice were plated and pretreated with 0 or 30 μM theaflavins for 12 h, followed by 5 ng/mL LPS and 0.5 ng/mL interferon (IFN)-γ for 12 h. Quantitative live cell imaging was performed to assess the length of the dendrites in the Neuro2A cells using an IncuCyte Zoom real-time imaging system (Essen Biosciences, Ann Arbor, MI, USA). Images were obtained every 24 h at 20× magnification in the phase-contrast mode. The length of the neuronal dendrites per neuron was determined using IncuCyte Neurotrack Software (Essen Biosciences). Each sample was assayed in three wells.

### 4.8. Statistical Analysis

Data are presented as the mean with error bars indicating the standard error (SE). Data were analyzed by Student’s *t*-test and one-way analysis of variance (ANOVA) followed by Dunnett’s test, as indicated in the figure legends. All statistical analyses were performed using the Ekuseru–Toukei 2012 software program (Social Survey Research Information, Tokyo, Japan). A *p*-value < 0.05 was considered statistically significant.
